# Glucoregulatory Properties of a Protein Hydrolysate from Atlantic Salmon (*Salmo salar*): Preliminary Characterization and Evaluation of DPP-IV Inhibition and Direct Glucose Uptake In Vitro

**DOI:** 10.3390/md22040151

**Published:** 2024-03-28

**Authors:** Christian Bjerknes, Sileshi Gizachew Wubshet, Sissel Beate Rønning, Nils Kristian Afseth, Crawford Currie, Bomi Framroze, Erland Hermansen

**Affiliations:** 1 Hofseth Biocare ASA, Keiser Wilhelms Gate 24, 6003 Ålesund, Norway; cc@hofsethbiocare.no (C.C.); bf@hofsethbiocare.no (B.F.); ehe@hofsethbiocare.no (E.H.); 2 Nofima AS, Osloveien 1, 1433 Ås, Norway; sileshi.wubshet@nofima.no (S.G.W.); sissel.beate.ronning@nofima.no (S.B.R.); nils.kristian.afseth@nofima.no (N.K.A.); 3 Faculty of Medicine and Health Sciences, Norwegian University of Science and Technology (NTNU), Larsgårdsvegen 2, 6009 Ålesund, Norway

**Keywords:** marine protein hydrolysate, DPP-IV inhibition, glucose uptake, glucoregulatory peptides, bioactive peptides, salmon protein hydrolysate, metabolic disease

## Abstract

Metabolic disorders are increasingly prevalent conditions that manifest pathophysiologically along a continuum. Among reported metabolic risk factors, elevated fasting serum glucose (FSG) levels have shown the most substantial increase in risk exposure. Ultimately leading to insulin resistance (IR), this condition is associated with notable deteriorations in the prognostic outlook for major diseases, including neurodegenerative diseases, cancer risk, and mortality related to cardiovascular disease. Tackling metabolic dysfunction, with a focus on prevention, is a critically important aspect for human health. In this study, an investigation into the potential antidiabetic properties of a salmon protein hydrolysate (SPH) was conducted, focusing on its potential dipeptidyl peptidase-IV (DPP-IV) inhibition and direct glucose uptake in vitro. Characterization of the SPH utilized a bioassay-guided fractionation approach to identify potent glucoregulatory peptide fractions. Low-molecular-weight (MW) fractions prepared by membrane filtration (MWCO = 3 kDa) showed significant DPP-IV inhibition (IC_50_ = 1.01 ± 0.12 mg/mL) and glucose uptake in vitro (*p* ≤ 0.0001 at 1 mg/mL). Further fractionation of the lowest MW fractions (<3 kDa) derived from the permeate resulted in three peptide subfractions. The subfraction with the lowest molecular weight demonstrated the most significant glucose uptake activity (*p* ≤ 0.0001), maintaining its potency even at a dilution of 1:500 (*p* ≤ 0.01).

## 1. Introduction

Metabolic disorders entail a fundamental malfunction in the body’s processing of nutrients, often stemming from an imbalance in nutrient intake, particularly in terms of excessive caloric consumption. In the 21st century, metabolic disease poses a substantial challenge to public health, fueled by a relentless rise in its incidence. An important downstream manifestation of metabolic disorders is related to glucose metabolism. Today, over 11% of the US adult population has clinical type 2 diabetes (DM2) according to a 2022 Center of Disease Control report [1]. Although the pathophysiology of type 2 diabetes (DM2) and metabolic syndrome differs, insulin resistance serves as a central metabolic derangement in both conditions.

A common early sign of metabolic disruption is elevated insulin levels. Continuous hyperinsulinemia can result in a cluster of metabolic abnormalities referred to as metabolic syndrome (MetS). This syndrome is defined somewhat arbitrarily by a collection of metabolic abnormalities including hypertension, elevated triglycerides, hyperglycemia, low HDL cholesterol, and central obesity [2]. Fulfilling three or more criteria establishes the presence of clinical MetS. While obesity is a criterion, it does not universally define metabolic unhealthiness, as not all metabolically unhealthy individuals are obese [3]. Addressing insulin resistance through therapeutic interventions is crucial, as research has identified it as a significant factor in developing chronic diseases. Several chronic diseases, such as cardiovascular disease, cancer, and neurodegenerative disease, are prognostically worsened by the presence of such metabolic abnormalities [4,5,6,7,8,9].

High fasting plasma glucose (HFPG) is the metabolic risk factor that has undergone the most substantial increase over time [10]. HFPG induces compensatory hyperinsulinemia, which, over the long term, can result in insulin signaling defects in tissues normally responsive to insulin [11]. Chronically elevated plasma glucose cause long-term negative effects on various tissues, in large part from glucose being irreversibly and non-enzymatically bound to proteins, lipids, or nucleic acids, leading to the formation of advanced glycation end products (AGEs) [12]. The presence of AGEs increases the likelihood of inappropriate activation of inflammatory and oxidative stress pathways, which contributes to negative health consequences [13]. A practical, easily accessible method involving the use of nutraceuticals or functional foods would be an appealing means to attempt to both delay and prevent the onset and progression of chronic metabolic diseases. 

Natural marine bioactive compounds have become a focus of highly intensive research in recent years, with researchers having successfully discovered and isolated over 12,000 novel metabolites [14]. Indeed, marine bioactive compounds and their health effects rank among the most extensively researched compounds in the last two decades [15,16]. Marine organisms that were once regarded as a source of nutrition, like the sea cucumber, are now viewed as reservoirs of valuable bioactive compounds that hold the potential to serve as therapeutics and drug candidates relevant in addressing human diseases [17]. A compelling category of marine bioactive compounds comprises bioactive peptides. Typically spanning from 3 to 20 amino acids in length, these functional fragments may influence physiological processes upon consumption, serving as active biological regulators. Bioactive peptides, often natural constituents of food, are found encrypted and inactive within a specified parent protein, becoming bioactive upon release. Fish-derived marine bioactive peptides and their by-products are typically obtained through biotechnological techniques, commonly enzymatic hydrolysis. This method involves a gentle manufacturing process in which enzymes break down proteins to produce a mixture of peptides, known as a protein hydrolysate [18,19]. Hydrolysates are crude mixtures of low-molecular-weight (MW) bioactive and non-bioactive peptides [20]. Results from one clinical trial demonstrated meaningful antihypertensive effects from shrimp-derived hydrolysates for individuals with mild-to-moderate hypertension. The physiological effects of bioactive peptides contained within protein hydrolysates are remarkably diverse, with some exhibiting multifunctionality [21]. Marine organisms exhibit various bioactivities relevant to metabolic pathways, encompassing glucoregulatory, blood pressure-regulating, anti-inflammatory, and antioxidant effects [22,23]. Notably, however, research on their potential bioactivities is ongoing and includes several other areas of investigation, including the immune system and aging processes [23]. These diverse effects, along with inherent attributes such as target specificity and general safety, have intensified researchers’ interest in their development. Sustainability concerns have shifted research towards the utilization of fish processing discards, now regarded as secondary raw materials, in the development of bioactive peptides and production of value-added protein hydrolysates that are suitable for human consumption [24]. A common occurrence with the enzymatic hydrolysis of various fish species and their processing discards is the generation of glucoregulatory bioactive peptides capable of interacting with enzymes involved in glucose homeostasis [25,26]. 

**Figure 1:**
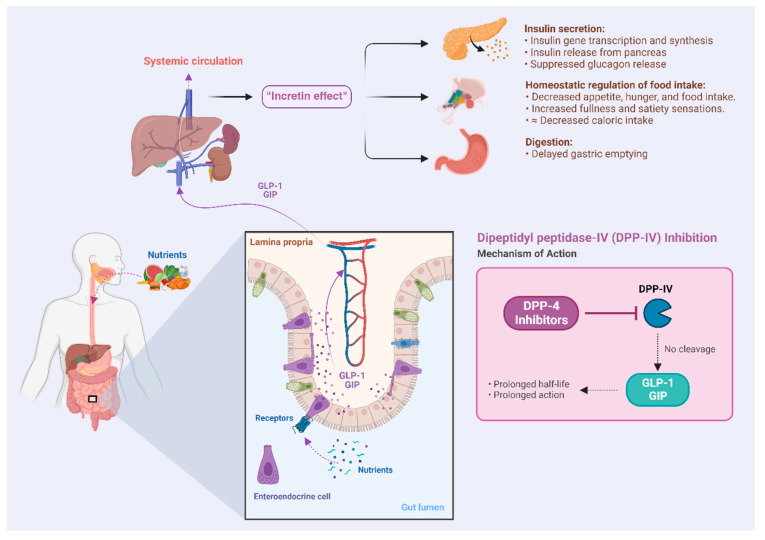
Illustration of the “incretin effect”, which refers to the enhancement of insulin effectiveness due to the post-prandial secretion of incretins from intestinal enteroendocrine cells.

Bioactive peptides liberated from Atlantic salmon (*Salmo salar*) are commonly observed to inhibit dipeptidyl peptidase IV (DPP-IV), and to exert various antioxidant effects [27]. DPP-IV inhibition prevents the breakdown of glucagon-like peptide 1 (GLP-1) and glucose-dependent insulinotropic polypeptide (GIP) that are released post-prandially, prolonging their half-life and amplifying the insulin effect on glucose homeostasis (Figure 1) [28]. Prolonged systemic circulation of incretins enhances the transcription, synthesis, and exocytosis of insulin from pancreatic islet cells, which constitutes the mechanism of amplifying the insulin response. Indeed, gliptins, a category of medications inhibiting DPP-IV enzymes, are drugs that exploit this mechanism in the management of type 2 diabetes (DM 2). Bioactive peptides targeting DPP-IV are typically derived from proline-rich motifs of collagenous proteins, such as those found in the skin of salmon. As shown by Harnedy and colleagues, significant DPP-IV inhibitory potential was demonstrated for a salmon-derived hydrolysate manufactured from processing discards [29]. Today, various commercially available marine protein hydrolysates based on processing discards are available. Similarly, Li-Chan and colleagues reported considerable DPP-IV inhibitory effects of a specific peptide, GPAE, derived from salmon gelatin, a partially hydrolyzed protein source [30]. Other glucoregulatory effects were demonstrated by Roblet and colleagues, showing enhanced glucose uptake in muscle cells in vitro following exposure to a salmon-frame hydrolysate [31]. Hydrolysates from other fish species, such as sardine [25], silver carp [26], and shark liver [32], have also been shown to exhibit diverse glucoregulatory properties. Animal research has yielded encouraging findings, indicating that interventions lasting six weeks with the use of hydrolysates demonstrate promise in alleviating insulin resistance [33]. Early indications from clinical trials have indicated that marine hydrolysates can have clinically beneficial metabolic effects [34,35,36].

In prior investigations involving a salmon protein hydrolysate (SPH) derived from the processing discards of Norwegian Atlantic salmon (*Salmo salar*), some of the authors of the current paper demonstrated favorable changes in various metabolic biomarkers in small-scale clinical trials in response to daily SPH administration. These trials also uncovered favorable changes in body mass index (BMI). A noteworthy observation was the observation of significant improvements in hemoglobin and serum ferritin levels, in the absence of supplementary or additional dietary iron. A small, yet consistent reduction (ranging from 3% to 6%) in fasting plasma glucose has also been observed [37,38]. Further in vitro investigations into the SPH have revealed significant fold-change increases in gene expression, particularly genes that are recognized as central for antioxidant defense mechanisms, specifically HMOX, a change that was accompanied by the downregulation of proinflammatory genes, including ALOX12 [39]. Transcription and translation of ALOX12 yields a lipoxygenase involved in proinflammatory metabolite pathways, which may be one aspect contributing to pancreatic islet cell inflammation and injury, which is seen in the pathogenesis of DM [40,41,42].

The objective of this study was to assess whether the SPH housed bioactive peptides capable of exerting glucoregulatory properties. Glucoregulatory potential was assessed in terms of capacity for DPP-IV inhibition and glucose uptake in rat myocytes in vitro. Initial characterization of the SPH regarding its molecular weight and peptide composition was conducted to generate foundational knowledge about its composition. Bioassay-guided fractionation technology is employed in further developments in the presence of the specified bioactivity.

The overarching goal of this research is to advance the development of an SPH into a readily accessible and practical nutritional strategy that could potentially promote healthy metabolic function, properly substantiated by scientific publications.

This approach could represent a readily accessible, cost-effective, and easily implementable strategy, potentially offering broad benefits for human health in a sustainable manner. 

## 2. Results

### 2.1. Characterization and Fractionation of SPH

**Figure 2:**
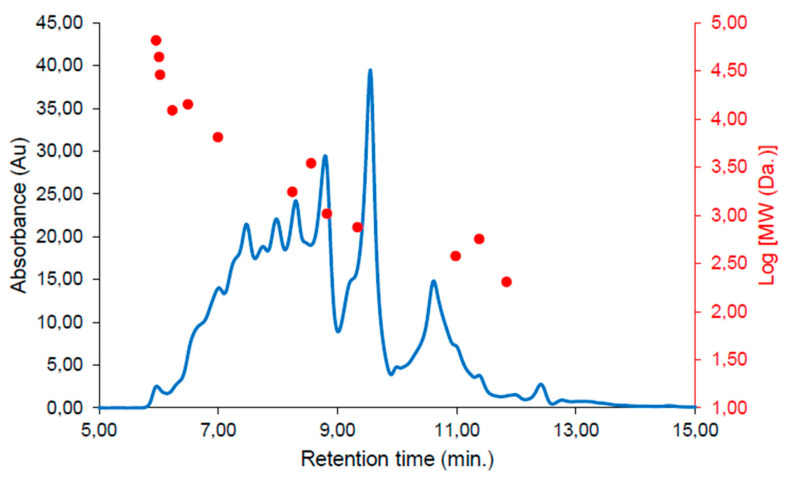
Size-exclusion chromatogram showing the molecular weight (MW) distribution of the SPH. The chromatogram was continuously monitored at 214 nanometers (nm). Correlations between the MW standards and retention time are presented as a dot plot overlayed in red.

Initially, peptides from the SPH were characterized by employing size-exclusion chromatography (SEC) and Fourier transform infra-red (FTIR) spectroscopy. SEC revealed the MW distribution of the crude SPH (Figure 2) to include peptides spanning a wide range of molecular weights. The average MW of the peptides for the crude SPH was calculated to be 3395 Daltons (Da).

**Figure 3:**
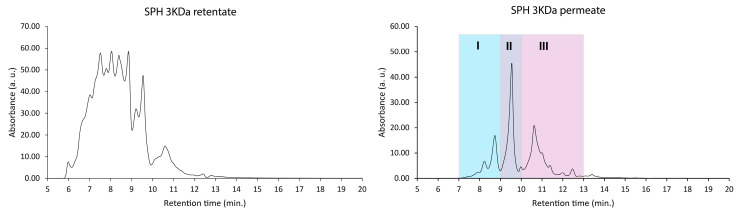
Molecular weight distributions (size-exclusion chromatogram) of the retentate (>3000 Da) and permeates (<3000 Da). Note that larger peptides and proteins elute earlier than smaller ones (see calibration data in Figure 2).

As an initial fractionation protocol, filtration with a MW cut-off threshold of 3000 Da was employed. Both permeates (MW < 3 kDa) and retentates (MW > 3 kDa) were collected post-fractionation and analyzed further using SEC (Figure 3).

The retentate fraction, representing the portion of the SPH retained by the filtration membrane, exhibited an average MW of 4109 Da. Comparably, the permeate, which denotes the portion of the SPH solution passing through the filtration membrane, displayed a substantially lower average MW of 880 Da.

**Figure 4:**
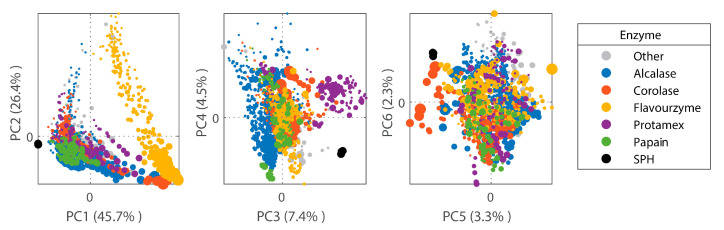
Scores of the first six FTIR-PCA components in a model based on 1300 protein hydrolysates. The dot size is proportional to hydrolysis time. Color represents employment of different enzymes in the manufacturing of a given hydrolysate (Alcalase represented by blue; Corolase represented by red; Flavourzyme represented by yellow; Protamex represented by purple; Papain represented by green). SPH samples (represented by black dots) were determined to be distant/unique from most of the database hydrolysates used for comparison.

In addition to SEC, FTIR fingerprinting was used in order to benchmark the peptide composition of the SPH against a previously published database of laboratory- and industrial-scale hydrolysates [43]. The FTIR fingerprint of the SPH was projected onto a Principal Component Analysis (PCA) model based on a database containing 1300 registered hydrolysates. The FTIR fingerprint of the SPH shows that its peptide composition constitutes a relatively unique peptide profile, placing it relatively distant from most hydrolysate samples in the database (Figure 4). This can be attributed to both differences in the enzyme used (i.e., PC1) and raw material differences (i.e., PC3 and PC4) [43].

### 2.2. Direct Glucose Uptake In Vitro

**Figure 5:**
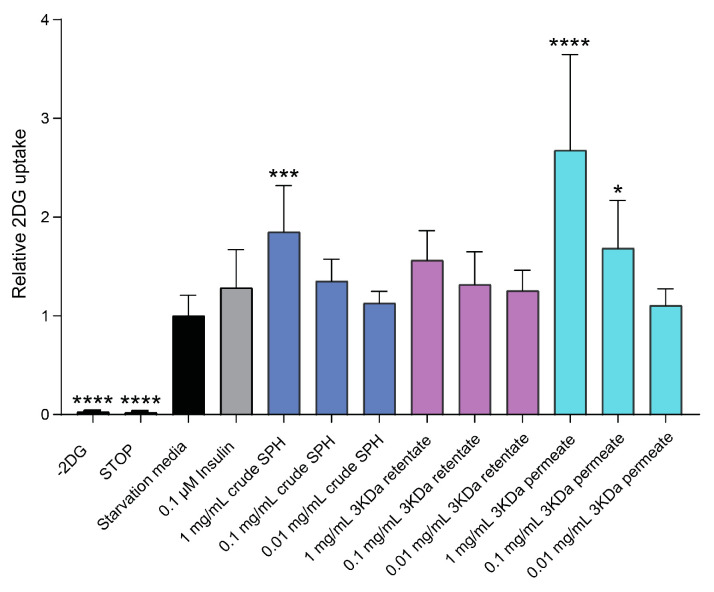
Effects of crude SPH, permeate, and retentate solutions on glucose uptake in L6 rat skeletal muscle cells in vitro. Bar plot of relative glucose uptake in cells treated with insulin or hydrolysates at indicated concentrations compared to control cells (i.e., untreated cells). The data are presented as the average of a cell culture experiment seeded out in triplicates ± SD. Asterisks denote significant differences (* *p* < 0.05, *** *p* < 0.001, **** *p* < 0.0001) compared to the control cells, calculated by one-way ANOVA using Dunnett’s multiple comparison test.

The effects of varying concentrations of crude SPH, permeate, and retentate are illustrated in Figure 5. L6 rat skeletal muscle cells treated with both crude SPH and 1.0 mg/mL permeate solution demonstrated significant increases in glucose uptake (*p* ≤ 0.001 and *p* ≤ 0.0001, respectively). Statistically significant effects were maintained at permeate concentrations of 0.1 mg/mL (*p* ≤ 0.05). The permeate, consisting of smaller MW peptides, was the primary driver of this effect on glucose uptake. No significant effect in terms of glucose uptake was observed for cells treated with retentate solutions, composed of higher-MW peptides. 

**Figure 6:**
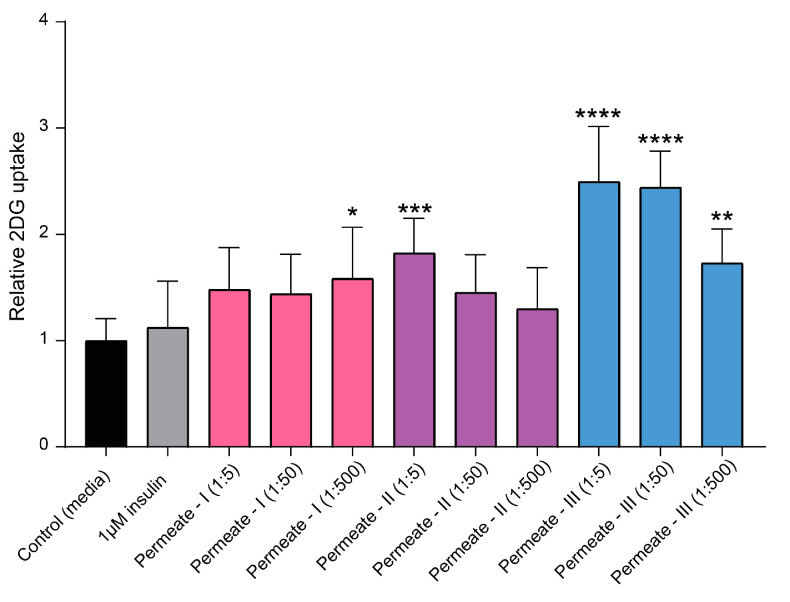
Effects of the subfractions permeate I, permeate II, and permeate III on insulin-independent glucose uptake in L6 rat skeletal muscle cells in vitro. Bar plot of relative glucose uptake in cells treated with insulin or hydrolysates at indicated concentrations compared to control cells (i.e., untreated cells). The data are presented as the average of a cell culture experiment seeded out in triplicates ± SD. Asterisks denote significant differences (* *p* < 0.05, ** *p* < 0.01, *** *p* < 0.001, **** *p* < 0.0001) compared to the control cells, calculated by one-way ANOVA using Dunnett’s multiple comparison test.

To identify the most bioactive peptide fraction within the permeate, the permeate mixture was further fractionated using SEC (see Figure 3; colorized chromatogram). The resulting three subfractions, denoted as permeate I, permeate II, and permeate III, were composed of peptides in decreasing order of MW. The permeate III subfraction, comprising the smallest peptides, exhibited the highest activity in terms of directly facilitating glucose uptake into muscle cells (*p* ≤ 0.0001; Figure 6). This subfraction exhibited a significant (*p* ≤ 0.01) increase in glucose uptake even at the lowest tested concentration (fraction diluted 1:500), indicating a potent bioactive potential. 

### 2.3. DPP-IV Inhibition

Crude SPH, permeate, and retentates were screened for their glucoregulatory properties in terms of DPP-IV inhibition. 

**Figure 7:**
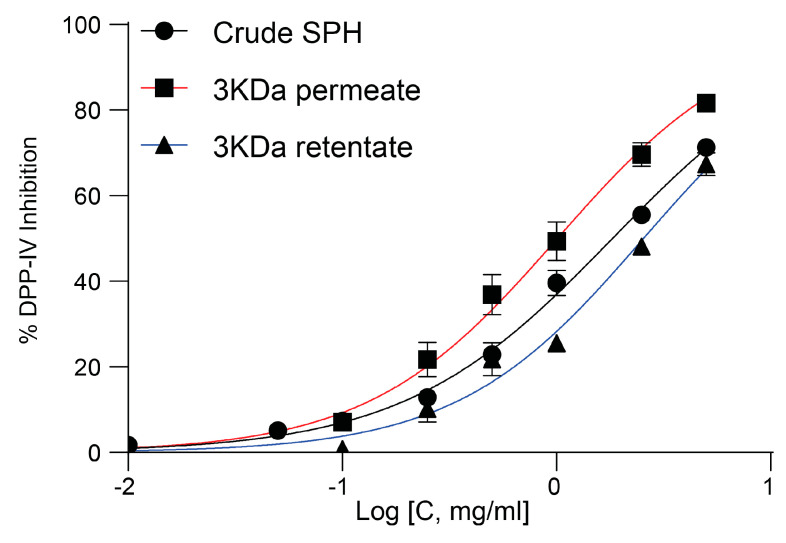
Dose–response curve illustrating DPP-IV inhibition activity of crude SPH, permeate, and retentate. Dose–response curves (left) and fitting results from IC_50_ value calculations (Table 1).

A capacity for DPP-IV inhibition was observed in all three types of samples, with the permeate specifically demonstrating the highest level of activity determined at an IC_50_ of 1.01 ± 0.12 mg/mL (Figure 7).

### 2.4. Liquid Chromatography–Mass Spectrometry (LC-MS) Peptide Identification

**Figure 8:**
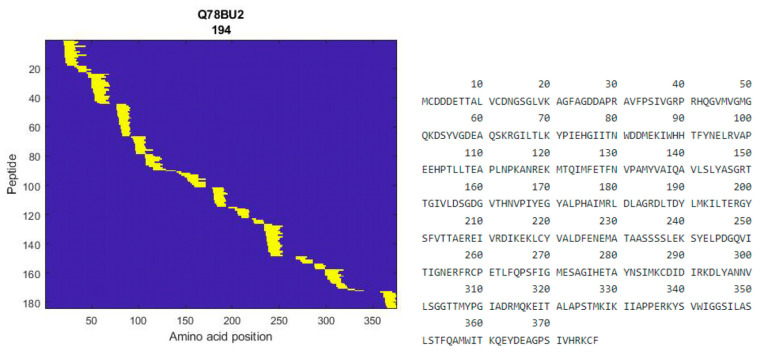
Heatmap of identified peptides in the permeate from alpha fast skeletal muscle actin from *Salmo salar* (**left**), and protein sequence of alpha fast skeletal muscle actin (**right**).

The fraction showing the most potential in terms of DPP-IV inhibition and glucose uptake effects underwent further analysis using MS-based peptidomics to enhance characterization and identify peptides. A total of 260 peptides were identified with a high “identification score”. A complete list of the peptides with the details of the identification is available as supplementary data (See Supplementary Materials: 260 peptides). As an example, one of the proteins that several peptides were identified as deriving from was alpha fast skeletal muscle actin from *Salmo salar* (Figure 8).

## 3. Discussion

In recent years, there has been a growing number of studies aiming to characterize and evaluate the feasibility and potential applicability of bioactivities in marine protein hydrolysates [44]. Marine hydrolysates are widely acknowledged as safe nutritional interventions, offering preventive and supportive approaches for managing metabolic disorders. Findings from clinical trials involving DPP-IV inhibitory hydrolysates have shown promising preliminary results, including improvements in fasting blood glucose (FBG) levels and glycosylated hemoglobin A1c (HbA1c) after three months of intervention [34]. Hence, the considerable health-promoting potential and feasibility of their utilization should warrant continued research efforts. On a population level, even modest improvements in a person’s metabolic risk profile would likely lead to significant reductions in overall human disease burden and healthcare costs. As a component of a comprehensive strategy to address the rising prevalence of metabolic diseases, focusing research efforts on the development of nutraceutical interventions emerges as a valuable pursuit. This study aimed to collect initial insights into the potential metabolic advantages of an SPH, alongside conducting a characterization of this hydrolysate in terms of peptide composition.

Upon initial characterization, the SPH exhibited a peptide composition distinct from that of 1300 commercial and laboratory hydrolysates listed in a recently published database. This distinctiveness was apparent in the distribution of peptide sizes, a characteristic primarily influenced by predefined manufacturing parameters such as the enzymes, hydrolysis time, and composition of raw materials employed. The average MW was 3395 Da, comprising a broad spectrum of peptide sizes. The permeate, containing the smallest peptide fraction, demonstrated the highest DPP-IV inhibitory potency, with an IC_50_ of 0.8983 to 1.137 mg/mL, consistent with results from some published DPP-IV assay experiments on hydrolysates [45,46,47], with some studies reporting a comparably lower DPP-IV inhibitory capacity 20–30%) than the SPH [30,48]. We found that this activity was mediated by the peptide fraction composed of smaller-sized peptides with an average MW of 880 Da. Indeed, this aligns with results from Zhang et al., where the most potent inhibitors were seen for the peptide fractions lower than 3 kDa [49,50]. From the literature available over the last five years concerning purified DPP-IV inhibitory bioactive peptides, the majority were identified as di-, tri-, and oligopeptides, indicating peptides of smaller sizes [51]. Reportedly, inhibitors with smaller molecular weights exhibit superior performance as DPP-IV inhibitors compared to larger-sized peptides [52]. The permeate, comprising peptides with an average molecular weight of less than one kDa, aligns with the literature which suggests that smaller peptides exhibit superior DPP-IV inhibition activity compared to higher-molecular-weight fractions [53,54,55]. Moreover, the permeate surpassed both the retentate and crude SPH in terms of directly enhancing glucose uptake into rat myotubules. Glucose uptake was increased by approximately 2.5-fold compared to control conditions, and 2-fold compared to insulin at concentrations of 0.1 µM, a common in vitro dose for such assays. [56]. Through additional bioassay-guided fractionation of the permeate, the fraction containing the smallest peptides, designated as permeate-III, exhibited a significantly greater capacity for glucose uptake compared to the original permeate. This is in line with previously published reports, showing that glucose uptake was improved in muscle cells when stimulated with low MW peptides from soy and poultry by-products [31,57]. A mechanism mediating and facilitating glucose uptake seems to be a commonly occurrence for salmon-derived peptide fractions [58]. Even when diluted to a ratio of 1:500, the potency of permeate-III in achieving statistically significant effects was maintained for this low molecular weight fraction.

The findings presented here reveal the presence of glucoregulatory peptides within a potent subfraction derived from an SPH with a peptide composition unique from that of other industrial hydrolysates. The more potent permeate, consisting of smaller peptides with an average MW of 880 Da, suggests that shorter peptides are the main mediators of this bioactivity. Applying the average MW of the 20 common amino acids, this corresponds to a peptide chain of 8 amino acids, which is consistent with the characteristic chain length for DPP-IV inhibitory peptides [59]. Whether these are specific peptides demonstrating dual glucoregulatory properties or distinct peptides each performing a unique function is yet to be determined. Notably, one of the most intriguing aspects of bioactive peptides is their potential to be multifunctional, with a single peptide capable of exerting more than one effect. By targeting multiple pathways involved in glucose regulation, synergy may potentially offer enhanced efficacy in managing metabolic disruptions. Glucose can be transported into different tissues either by sodium-dependent glucose co-transporters, which do not depend on insulin, or by specialized glucose transporters known as GLUTs. The precise mechanism through which the presence of peptides facilitates glucose uptake remains to be understood. We can hypothesize whether GLUT-4 translocation to the plasma membrane might be responsible for some of the effect, occurring through the phosphoinositide 3-kinase pathway (PI3K-Akt) [46,60]. Bioactive peptides are reported to increase glucose uptake through both insulin-dependent and -independent pathways. Glucose uptake through insulin-independent pathways, specifically, frequently involves the activation of AMP-activated protein kinase (AMPK), presenting potential avenues for additional investigations for the SPH [61]. In a previous study, low-MW peptide fractions from soybean demonstrated enhanced glucose uptake in L6 muscle cells when insulin was present [31]. Although these peptides were found to activate AMPK, glucose uptake was not enhanced in the absence of insulin.

Potent glucoregulatory properties were also evident for the lower-MW peptide fraction in terms of DPP-IV inhibitory activities. The mechanism of enzyme inhibition, whether it involves competitive, uncompetitive, or non-competitive pathways, or even mixed modes by binding at either the active site and/or outside the catalytic site of the DPP-IV enzyme, awaits further clarification in future studies. Further investigations of the glucoregulatory properties described herein should consider fractionation as an initial measure to concentrate sufficient quantities of the potent low-molecular-weight constituents. In an initial peptide screening using LC-MS peptidomics, 260 peptides with unique sequences ranging from 8 to 25 amino acids in length were identified as highly likely to be bioactive and to exert relevant glucoregulatory effects. From the peptides derived from alpha fast skeletal muscle actin, we notice structural repeats in certain peptides, such as GP and PG sequences, which are common structures for DPP-IV inhibitory peptides [62]. Additionally, we observe proline flanked by leucine or valine residues, LP and VP, in some of the peptides, as a frequently reported feature in DPP-IV inhibitory peptides [25,63]. We can gain some insights into this specific peptide composition using predictive peptide tools. The list of 260 peptides was evaluated with an online predictive tool, Peptide Ranker, which employs neural networks to evaluate bioactivity based on peptide primary structure. A rank of 0.0 represents high likelihood of no bioactivity being present, while a rank of 1.0 indicates the very highest likelihood of bioactivity present [64]. The prediction tool does not make predictions in terms of the nature of any given peptide, merely whether it is likely to be bioactive or not bioactive. A compilation of ranked peptides can be found in the supplementary materials (see Supplementary Materials: Ten Ranked Peptides). A rank threshold of 0.8 was chosen to filter only the peptides most likely to be bioactive, which affords a false positive rate of 6% for small peptides as reported by Bioware [65]. A total of 10 peptides out of the 260 listed peptides were assigned a rank higher than 0.8, suggesting a high likelihood of bioactivity with a corresponding low false positive rate, with lengths from 13 to 20 amino acids long. The ten ranked candidate peptides are seen harboring an abundance of Gly-Pro-type peptides, which may have been liberated from various types of collagen derived from the skin, head, and trimmings of the Atlantic salmon (*Salmo salar*) raw materials. Collagen is notably abundant in proline triplets, where proline typically occupies position 2 of these triplets, typically denoted as Gly-X-Y. Here, X and Y can represent any amino acid, although proline or hydroxyproline residues are typical [66]. Proline residues in the second position from the N-terminus are indeed a common structural feature of DPP-IV inhibitory bioactive peptides, a characteristic shared by every ranked peptide [67]. The peptides listed can be expected to exhibit varying degrees of DPP-IV inhibition based on this, with their potency likely influenced by the remaining portions of their primary sequences. Enzymatic hydrolysis of Atlantic salmon (*Salmo salar*) skin has produced peptides akin to the nine ranked peptides identified by Peptide Ranker, albeit shorter in length. Specifically, sequences reported included GPAE and GPGA, which exhibited potent IC_50_ values in terms of DPP-IV inhibition [30]. A glycine–proline type of peptide cannot, however, be a mandatory feature of peptides with DPP-IV inhibitory capacities, as novel peptides liberated from Atlantic salmon gelatin have yielded the peptide LDKVFR, which was determined to be quite potent [68]. Hydrophobic interactions of the peptide backbone with its DPP-IV target enzyme, along with other forces such as Van der Waals interactions, are those that would dictate how the peptide interacts with and modulates its target. The specific features contributing to the very highest potency of marine-derived DPP-IV inhibitory peptides, including their length and primary sequence, have not been fully elucidated at present.

The current findings are promising. Atlantic salmon (*Salmo salar*) processing by-products, often considered secondary raw materials in the production of value-added human-consumable products, are frequently identified as potent sources of glucoregulatory peptides. In one pre-clinical study on knockout mice, a fish protein hydrolysate was deemed to exert a capacity for beneficial glucoregulatory effects, with a potency on par with Metformin, a drug used in the management of type 2 diabetes [69]. A crucial future consideration is the bioavailability of such interventions, should such interventions be employed to address metabolic abnormalities. Certainly, addressing bioavailability remains crucial for ensuring the acceptance and effectiveness of purified hydrolysate interventions. At the same time, smaller peptides tend to exhibit greater resistance to gastrointestinal proteolysis, which may allow them to be absorbed into the circulation in an intact form [70,71]. The ranked peptides cannot be classified strictly as short peptides, however, should they become the most promising peptide candidates.

Even if the metabolic benefits of regular consumption of bioactive hydrolysates are modest, in the case of bioavailability limitations, the overall effects would be highly favorable on a population-wide scale. Another consideration is determining the extent of purification employed for a hydrolysate to enhance its glucoregulatory potency, while ensuring that this level of purification is still cost-effective and feasible for industrial-scale manufacturing. Additional in silico and in vitro investigations are necessary next steps to pinpoint the most potent and promising peptide candidates. These peptide candidates will subsequently undergo further characterization, including molecular docking investigations. Utilizing salmon raw materials sustainably to manufacture products with potentially meaningful health benefits appears to be an appealing and straightforward developmental trajectory. Since these peptides originate from food sources, they tend to be inherently safe for human consumption. In terms of conducting a comprehensive exploration of potential antidiabetic mechanisms, it would be intriguing to examine the possible presence of incretin mimetics within the SPH, as has been demonstrated for some hydrolysates [72]. Purified peptides will be further employed in appropriate pre-clinical animal models to assess their efficacy in an in vivo environment.

## 4. Conclusions

The current study provided initial characterization and bioactivity screening of a protein hydrolysate derived from Atlantic salmon (*Salmo salar*) raw materials. The initial analysis indicated that the SPH comprises relatively large peptides, with an average size of 3395 Da. The FTIR spectrum expressed a distinct signature compared to a large library of industrial hydrolysates, suggesting a unique peptide composition. 

Bioactivity assaying unveiled potent glucoregulatory properties, operating through two distinct mechanisms. The permeate exhibited the highest potency, indicating that the glucoregulatory activity is mediated primarily by short-chained bioactive peptides. LC-MS-driven peptidomics identified more than 260 relevant peptides in the permeate, with 10 predicted to possess the highest bioactivity. The primary structures of these ten peptides displayed features commonly found in DPP-IV inhibitory peptides. 

Biotransformation of salmon processing discards offers high-quality nutrition and potent glucoregulatory peptides, which, if applied on a population scale, could meaningfully alleviate the growing health burden of metabolic disease. Bioactive peptides emerge as promising and versatile biological tools suited for use in future personalized medicine.

A feasible and safe peptide intervention for general use, whether as crude hydrolysates or permeates offering health benefits, or as highly purified bioactive fractions, will require distinct considerations. Randomized clinical trials and bioavailability assessments are essential prerequisites to guarantee their successful application.

## 5. Materials and Methods

### 5.1. Manufacturing Salmon Protein Hydrolysate

The head, skin, trimmings, frames, and bones (regarded as secondary raw materials) of Atlantic salmon (*Salmo salar*), arriving from supplier-audited fileting plants, are received at the hydrolysis plant on a daily basis. Secondary raw materials are then ground up and directed into a hydrolysis tank after which food-grade enzymes, Foam Control 30, and water are added to generate a mixture. The product is heated in two steps to a final temperature of 95 °C, held for a period of 5 min. The final mixture is transferred and split into respective fractions. Upon centrifugation, a final separation of the mixture into an SPH fraction, partially hydrolyzed protein (PHP) fraction, salmon oil fraction, and collagenous hydroxyapatite fraction is generated. Subsequently, the protein concentrate is sent through a heat exchanger, is homogenized, and finally, is spray dried. The product is sieved and packed. 

### 5.2. Sample Materials and Chemicals

SPH consists of a broad array of peptides derived from the enzymatic hydrolysis of proteins from salmon raw materials employing non-GMO protease enzymes. The degree of hydrolysis is estimated at 10%. The SPH appears as a light-yellow powder. It has a water-soluble protein content of >95%, of which >25% is composed of type I/III collagen peptides, with a fat content of <0.5%, and an ash content of <2.5%.

The amino acid composition is glutamic acid (13.9 g/100 g), aspartic acid (9.4 g/100 g), glycine (14.9 g/100 g), proline (7.6 g/100 g), lysine (7.0 g/100 g), alanine (7.5 g/100 g), and arginine (6.9 g/100 g). Hydroxyproline content was quantified to 4.18 g/100 g (ISO 13903:2005) [73]. Moreover, it contains meaningful amounts of vitamin B12 (cyanocobalamin), 27.9 µg/100 g (batch: sph 23012), as well as selenium (Se), 0.77 mg/kg (batch: sph 23012). Regular safety checks are performed by Eurofins as the process pertains to heavy metals, aflatoxins, dioxins, furans, and polychlorinated biphenyls. The SPH is screened negative for pesticides. The molecular weight peptide distribution is 5.5% for peptides <200 Da, 10.0% for peptides 200–500 Da, 14.1% for peptides 500–1000 Da, 20.4% for peptides 1000–2000 Da, 21.2% for peptides 2000–4000 Da, and 13.2% for peptides 4000–6000 Da, and remaining amounts are accounted for by higher-molecular-weight peptides.

Analytical grade acetonitrile, trifluoracetic acid (TFA), and monosodium phosphate used for size-exclusion chromatography (SEC) were purchased from Sigma-Aldrich (Oslo, Norway). A DPP4 inhibitor screening assay kit was purchased from Abcam (Amsterdam, Netherlands). Water was prepared by deionization and membrane filtration (0.22 μm) using a Millipore Milli-Q purification system (Molsheim, France).

### 5.3. FTIR-Based Fingerprinting and Benchmarking

Dry-film FTIR analysis was performed according to Wubshet et al. [74]. SPH was dissolved in ultrapure water to a concentration of 20 mg/mL, and 10 µL of such a sample was deposited onto a 96-slot Si-microtiter plate (Bruker Optik Gmbh, Germany) and dried at room temperature to form dry films. The sample was made in five replicates and measured by a High Throughput Screening eXTension (HTS-XT) unit coupled to a Tensor 27 spectrometer (both Bruker Optik Gmbh, Ettlingen, Germany). The spectra were recorded in the region between 4000 and 400 cm^−1^ with a spectral resolution of 4 cm^−1^ and an aperture of 5.0 mm. A second derivative was applied to the spectra using the Savitzky–Golay algorithm with a polynomial degree of two and a smoothing window size of 13 points. Similar to the database spectra published by Måge et al. [43], the spectra were normalized using a standard normal variate (SNV). For benchmarking, the FTIR fingerprints of SPH were projected into an in-house (Nofima, Ås, Norway) PCA model based on an FTIR spectral database of 1300 industrial and laboratory-produced hydrolysates, as described in Måge et al. [43]. 

### 5.4. Size-Exclusion Chromatography

The MW distributions of crude SPH, permeate, and retentate were analyzed with a size-exclusion chromatograph (SEC) according Wubshet et al. [74]. Briefly, 10 μL of the peptide solution (20 mg/mL) was separated in a BioSep-SEC-s2000 column (Phenomenex, Værløse, Denmark, 300 × 7.8 mm) coupled with a Dionex UltiMate 3000 HPLC system (Thermo Scientific, Waltham, MA, USA). The mobile phase was acetonitrile (30% (*v*/*v*)) in ultrapure water (70% (*v*/*v*)) containing 0.05% TFA. The flow rate was 0.9 mL/min, and the UV absorption was monitored at 214 nm. Molecular weight distribution and weight-average molecular weight (MW) of the crude hydrolysate and fractions were calculated using PSS winGPC UniChrom V 8.00 software (Polymer Standards Service, Mainz, Germany). Standards for calibration and calculations of molecular weight distribution and average MW were made as described in Wubshet et al. [74]. 

### 5.5. Filtration and SEC Fractionation

A solution of 10 mg/mL of SPH was prepared in ultra-filtered water and filtered through a Millex-HV PVDF syringe filter with a pore size of 0.45 mm (Merck Millipore, Billerica, MA, USA). The resulting crude SPH solution was subsequently fractionated using Amicon centrifugal filters, MWCO 3 kDa. The filtration procedure was carried out in accordance with the instructions provided by the manufacturer. Briefly, 15 mL (per single filtration) was loaded to the filters and centrifugation was performed at 4400 rpm for 20 min at 25 °C. Permeates and retentates from the filtration were collected and were freeze dried before further analysis.

Molecular-weight-based fractionation of the ProGo-permeate was performed using the same column and same method explained in Section 2.1 with higher loading. A total of three fractions were collected in the elution periods of 7–9 min (permeate I), 9–10 min (permeate II), and 10–13 min (permeate III). These three subfractions were freeze dried before further bioactivity evaluation.

### 5.6. Skeletal Muscle Glucose Uptake 

Cellular models are essential for studying biology in controlled settings. They offer detailed understanding of the molecular pathways and cellular reactions associated with both healthy and diseased states. In this context, L6 myotubes are particularly useful. Their pattern of GLUT (glucose transporter) expression closely resembles that of fully differentiated mammalian muscle. Specifically, they exhibit high levels of GLUT4 and relatively low levels of GLUT1 and GLUT3 [75]. L6 rat myoblasts (CRL-1458, ATCC) were maintained in high glucose containing DMEM with 10% FBS, 0.1% penicillin/streptomycin, and 0.1% Fungizone. Glucose uptake experiments were performed on differentiated L6 rat myotubes (CRL-1458, ATCC), initiated as follows: Amounts of 5000 cells per well were seeded out in a white 96-well plate with a flat bottom using a high-glucose DMEM (ATCC 30–2002) cell culture medium with 10% FBS, 0.1% penicillin/streptomycin, and 0.1% Fungizone. After 4 days of proliferation, differentiation was initiated using a differentiation medium with high-glucose DMEM (ATCC 30–2002) 2% FBS, 0.1% penicillin/streptomycin, and 0.1% Fungizone. The differentiation medium was changed every day for three days, before the myotubes were placed in a starvation medium, containing DMEM with no glucose, phenol red, and glutamine (A1443001, ThermoFisher), and 0.1% penicillin/streptomycin and 0.1% Fungizone. Differentiation was monitored in the microscope throughout the experiment. After 18 h in the starvation medium, myotubes were incubated in the presence or absence of insulin and hydrolysates for 1 h at the concentrations indicated in the figure legend. Control cells were left in the starvation medium.

**Figure 9:**
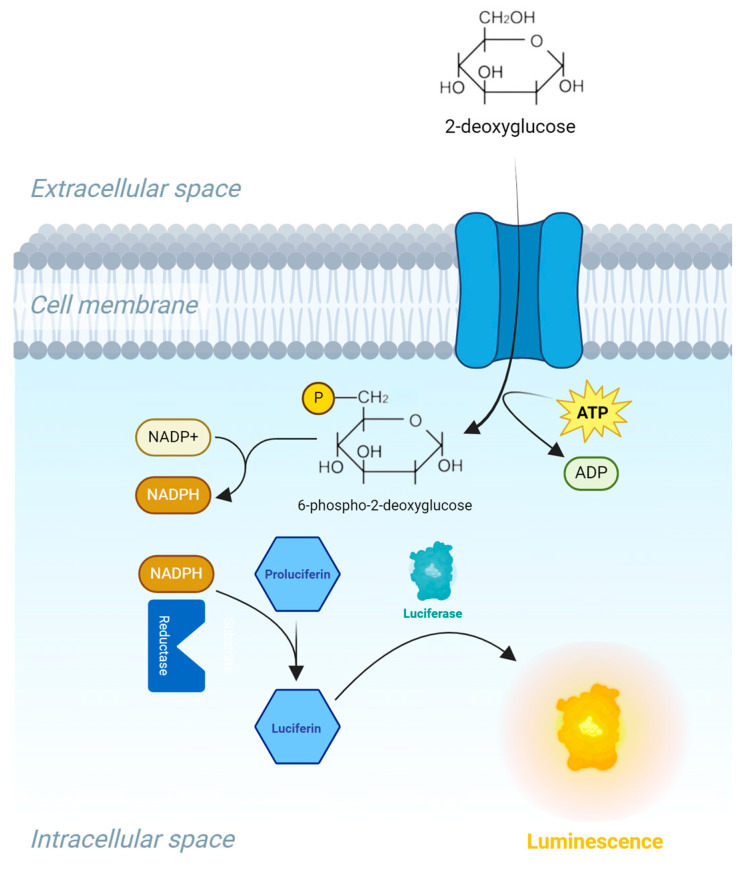
Illustration of the bioluminescence method for measuring glucose uptake in cells, based on the detection of 2-deoxyglucose-6-phosphate (2DG6P). First, 2DG is added to the cells, and is transported into the cell. Then stop and neutralization buffers are added to stop the reactions, lyse the cells, and eliminate the NADPH present inside the cells. The 2DG6P detection reagent is added; this will oxidize 2DG6P to 6-phosphodeoxygluconate and reduce NADP+ to NADPH. Proluciferin is then converted to luciferin, which acts as a substrate for a recombinant luciferase that produces a luminescence signal proportional to the concentration of 2DG6P.

Glucose uptake was performed using the Glucose Uptake-Glo™ Assay (J1342, Promega) according to the manufacturer’s instructions. In brief, cells pre-treated with insulin or hydrolysates for 1 h were washed with PBS, followed by 30 min incubation with 2-deoxyglucose (2DG). 2DG is transported across the cell membrane and undergoes intracellular phosphorylation, in the same manner as the breakdown of glucose during glycolysis. This process results in the formation of 2DG6P, which traps the substrate within the cell, leading to intracellular accumulation. However, due to steric hindrances, 2DG6P cannot be further processed in the glycolysis pathway. This allows for the assessment of 2DG uptake only. One hour after incubation with a 2DG6P detection reagent containing NADP+, reductase, and proluciferin enzymes, the intracellular 2DG6P is measured. The presence of 2DG6P reduces NADP+ to NADPH. This NADPH is then used by a reductase to convert proluciferin to luciferin. Luciferin serves as a substrate for luciferase, producing a luminescent signal proportional to the concentration of 2DG6P (See Figure 9). This allows for the quantification of 2DG uptake.

Luminescence was then recorded by a plate reader. Omitting 2DG or adding STOP buffer prior to 2DG incubation was used as negative controls. An amount of 0.1 µM insulin was employed as a positive control.

### 5.7. DPP-IV Inhibition

DPP-IV inhibition of crude SPH, permeate, and retentate fractions was evaluated using a commercial fluorometric dipeptidyl peptidase-IV (DPP-IV) inhibitor screening assay kit (Ab133081) produced by Abcam (Berlin, Germany). The protocol for the assay followed the instructions provided by the manufacturer. All three samples were tested in 8 different concentrations (from 0.01 mg/mL to 5.0 mg/mL). Test solutions were prepared in the assay buffer and all concentrations were tested in triplicates.

Fluorescence intensity was measured with a Microplate Reader Synergy H1 (BioTek, Winooski, VT, USA) using an excitation wavelength of 355 nm and an emission wavelength of 455 nm. Inhibition of DPP-IV (*I_DPP-IV_*) was calculated as follows:

$$I_{DPP\text{-}IV}\left(\%\right)=\left(\frac{initial\;activity-inhibitor}{intial\;activity}\right)\times 100$$

where *initial activity* is the activity of DPP-IV without the inhibitor present and *inhibitor* is the activity of DPP-IV with the sample present. In addition to the test samples, a positive control, sitagliptin, was tested in triplicate in the same plate. At a predetermined concentration provided with the assay kit, sitagliptin showed DPP-IV inhibition of 87.64% (±0.07).

The data fitting and IC_50_ value calculation were performed using GraphPad Prism (La Jolla, CA, USA).

### 5.8. Liquid Chromatography–Mass Spectrometry de Novo Sequencing of Peptides

Peptides were resolved with loading buffer [2% (*v*/*v*) CAN and 0.05% (*v*/*v*) trifluoroacetic acid]. Peptides were loaded onto a trap column (Acclaim PepMap 100, C18, 5 µm, 100 Å, 300 µm i.d. × 5 mm, Thermo Fisher Scientific) and then backflushed with a loading buffer described below onto a 50 cm × 75 µm analytical column (Acclaim PepMap RSLC C18, 2 µm, 100 Å, 75 µm i.d. × 50 cm, nanoViper, Thermo Fisher Scientific, Bremen, Germany) for LC-MS/MS analysis. Conditions for ultra-high-performance LC were as follows: loading pump, flow rate 20 µL/min with loading buffer; 2% (*v*/*v*) ACN and 0.05% (*v*/*v*) formic acid (FA) and nano/cap pump, flow rate 0.3 µL/min with a gradient of two buffers, A [0.1% (*v*/*v*) FA] and B [80% (*v*/*v*) CAN, 0.08% (*v*/*v*) FA]. The LC gradient was run for 120 min, from 3.2 to 80% buffer B. Peptides from the 12 most intense peaks were fragmented, and the mass-to-charge values of these fragmented ions were measured (tandem mass spectrometry, MS/MS) with a Q-Exactive Quadrupole-Orbitrap mass spectrometer (Thermo Fisher Scientific, USA). The Q-Exactive mass spectrometer was set up as follows: a full scan (300–1500 *m*/*z*) at R = 140,000 was followed by (up to) 12 MS2 scans at R = 17,500 using an NCE setting of 28. Singly charged precursors were excluded for MS/MS, as were precursors with z > 5. Dynamic exclusion was set at 20 s. Finally, peptide identification was performed using MaxQuant, an integrated suite of algorithms specifically developed for high-resolution, quantitative MS data. Raw LC-HRMS/MS data were searched against a non-specific digest of *Salmo salar* (*Atlantic salmon*) proteins (UniProtKB database). 

## Figures and Tables

**Table 1:** IC_50_ values for crude SPH, permeate, and retentate along with their respective 95% confidence intervals (CIs).

	Crude SPH	Permeate	Retentate
Best-fit values			
Log IC_50_	0.2613	0.004061	0.4062
Hill slope	0.8898	0.9853	0.9966
IC_50_	1.825	1.009	2.548
95% CI (profile likelihood)			
Log IC_50_	0.2350 to 0.2882	–0.04659 to 0.05563	0.3514 to 0.4647
Hill slope	0.84202 to 0.9421	0.8861 to 1.096	0.8746 to 1.137
IC_50_	1.718 to 1.942	0.8983 to 1.137	2.246 to 2.915

## Data Availability

Data requests may be made to the authors of this paper.

## Supplementary Materials

The following supporting information can be downloaded at: https://www.mdpi.com/article/10.3390/md22040151/s1, 260 Peptides, IC50 results raw and Peptide Ranker.
