# Supplementary material for: Glucoregulatory Properties of a Protein Hydrolysate from Atlantic Salmon (Salmo salar): Preliminary Characterization and Evaluation of DPP-IV Inhibition and Direct Glucose Uptake In Vitro
Source: Mar Drugs. 2024 Mar 28;22(4):151. doi: 10.3390/md22040151 (PMC11050766; doi:10.3390/md22040151)
Supplement: Supplementary file 1 [file marinedrugs-22-00151-s001.zip › Supplementary Material - Ten Ranked Peptides.pdf]

PeptideRanker

Your query (ID 171084754113809) has been processed (19.03.2024).

Response:

**0.964729**	**GPPGPPGPPGPPGPPGGGFD**
**0.930727**	**GPPGPPGSSGPQGF**
**0.909966**	**GPAGPNGPAGGRGGLGPA**
**0.899642**	**GPAGPHGPPGKDGRAGGHG**
**0.889048**	**GPRGPPGPPGSSGPQGF**
**0.873021**	**GPSGERGPPGPMGPPGLA**
**0.846923**	**GPPGPPGSSGPQG**
**0.819015**	**GPVGPAGKSGDRGESGPAGPA**
**0.809429**	**GPVGPAGKSGDRGESGPAGPAG**
**0.805951**	**PGPGGPQGEPGAVGPAG**
0.791772	MGPRGPPGPPGSSGPQGF
0.782283	LGPTGPIGTPGPMGPR
0.782179	GLPGGPGAVGEPGRLGPA
0.781425	GPAGPRGPSGPA
0.78115 KAADTFNFKTF	
0.778331	GPSGPAGARGDKGEAGEAGER
0.776344	GPPGPPGSSGPQ
0.771791	GPRGPPGPPGSSGPQG
0.759302	SSYGGANRGMGGGFGAGGGGGSY
0.750221	GASGPAGPRGPSGSAGAA
0.746106	PGPGGPQGEHGQIGPAG
0.740213	GPMGPRGPPGPPGSSGPQG
0.729904	GEPGKQGPGGPSGERGPPGPM
0.729846	GPSGPAGPSGESGPA
0.727939	SSGGQAQQGPMGPMGPR
0.722902	TGPPGEPGEAGSSGPMGPR
0.720204	SGESGPAGASGPAGPR
0.698244	SAGGGAGGAGAGAGGSVARGA
0.693342	GPTGPSGAPGFPGGAGAK
0.691649	GPRGPPGPPGSSGPQ
0.688174	GDRGDQGVKGADGAGGKDGIRG
0.683052	SGGGQAQMGPMGPMGPRG
0.680767	FTGPPGEPGEAGSSGPMGPR
0.666412	GPMGPRGPPGPPGSSGPQ
0.661017	PGPGGPQGEHGQIGPA
0.659388	GESGPAGPAGPNGPAGGRGGLGPA
0.65547 GPAGGQGEKGEGGPAGPN	
0.645763	GPMGPMGPRGPPGPPGSSGPQ
0.644583	GPPGPPGPPGPPGPPGGGFDM
0.639159	MGPRGPPGPPGSSGPQG
0.639116	PGPGGPQGEHGQIGP
0.638675	GEPGKQGPGGPSGERGPPGPMGPPG
0.630867	SGPAGPAGPNGPAGGRGGLGPA
0.618146	GPAGARGADGSTGPAGPAGPL
0.609093	RGPPGPPGSSGPQ
0.608775	GPAGAQGEKGEGGPAGPN
0.604673	GFTGPPGEPGEAGSSGPM
0.595796	GPAGARGADGSTGPAGPAGPLG
0.591814	GFTGPPGEPGEAGSSGPMGPR
0.581004	GEKGQPGGDGPNGPSGTPGPQ
0.577045	FTGPPGEPGEAGSSGPM
0.557906	GLTGPAGPQGARGFQGETGIQ
0.557057	MGPRGPPGPPGSSGPQ

0.547469	FSGLDGAKGETGPAGPKGEAG
0.535769	GMTGSPGSPGPDGKVGPA
0.5234	LRGDPGPGGPQGEHGQIGPA
0.521516	WAAFPPDVAGNVDYKQ
0.521043	GPAGSPGDKGETGAPGAVGPS
0.515051	GHGGLQGMPGPNGPSGETGSAG
0.505036	SGESGPAGASGPAGPRGPSGSAG
0.497159	GPAGARGADGSTGPAGPAGP
0.483323	NWDDMEKIWHH
0.482102	GESGPAGASGPAGPRGPSGSAGAA
0.480255	GSRGEPGNPGPAGAAGPS
0.475757	FSGLDGAKGESGPAGPKGEAGAS
0.47384	GTEASGTGNMKFM
0.473292	SGLDGAKGETGPAGPKGEAGAS
0.46315	GYTGATGATGLTGPAGPQGARGF
0.452053	FSDEEEFPDLSLH
0.451784	EALGDKAKFAGK
0.435222	GERGEQGAGGPPGFQ
0.419701	SGESGPAGASGPAGPRGPSGSAGAA
0.410298	STGEAGATGPAGLRG
0.409454	FSGLDGAKGETGPAGPKGEAGAS
0.40943	GARGADGSTGPAGPAGP
0.404232	AGPAGPAGARGADGSTGPAGPAGPL
0.403028	GLDASSGELPVVGIR
0.397305	KEYEPEMGKVF
0.395104	LPYDTPIPGYRNN
0.394118	ALPYDTPIPGYRNN
0.379334	GVVGNAGEKGEQGPSGAPGFQ
0.378089	LEQQVDDLEGSLEQEKKLRMD
0.377822	ATGATGLTGPAGPQGARGF
0.368547	LDGAKGESGPAGPKGEAGAS
0.367079	SMPGPMGPMGPRGPPGPPGSSGPQG
0.360745	LQGMPGPNGPSGETGSA
0.357719	PANIKWGDAGATY
0.35649	AGARGADGSTGPAGPAGP
0.346779	GEEGKRGSTGEVGATGPAGLR
0.345106	DAGKTFPQHVKSR
0.33901	GGRSFDNVDTPASAL
0.334044	HEDYKFGHTK
0.330889	GSDSLDSAKKEIG
0.328393	AGGGYDQSGGYDEYR
0.327242	GQKDSYVGDEAQSKRGIL
0.32248	DGAKGETGPAGPKGEAGAS
0.320068	DAGKTFPEHVKSR
0.31553	YGKDATNVGDEGGFAPNIL
0.306525	ITGDQLGDLYKSF
0.302948	GEQIDNLQRVK
0.301484	IITNWDDMEKIWHH
0.30112	FDMFDTDGGGDISTK
0.300186	EMNPPKYDKIEDM
0.298442	TGPAGPQGARGFQGETGIQ
0.295713	GFTNDEKIGIY
0.29555	DEYNKGFLDQL
0.292514	LDGAKGETGPAGPKGEAGAS
0.2883	AYEPVWAIGTGKTASPA
0.288296	AGPAGGGYDQSGGYDEY
0.283778	AADESTGSVAKRF
0.278917	GQKDSYVGDEAQSKRGILTL
0.278757	LRGHGGLQGMPGPNGPSGETGSAG
0.269936	LDGTENKSKFGAN
0.269248	AEELKKEQDTSSHLERMKKNLE

0.265862	GFTNEEKLGIYK
0.26388	DFNGDTHSSIFDAGAGIA
0.263431	IDSELPVDEVFGH
0.262138	AADGPMKGILGYTEQ
0.254358	EYDEAGPSIVHRK
0.253828	SSSSLEKSYELPDGQ
0.253661	SAETEKELANMK
0.25276	IIHGSDSLDSANKEIAL
0.249465	EISDLNRMIQ
0.249457	ATESFGPGTIEYRP
0.247899	AEELKKEQDTSSHLERM
0.247209	GQKDSYVGDEAQSKRGILT
0.239792	ELDGTENKSKFGAN
0.233679	GFTNEEKLGIY
0.233347	LGSIDVNHEDYKFGHTK
0.231868	EYDESGPSIVHRK
0.229719	SYEEALDHLETL
0.225556	GEQIDNLQR
0.225257	SEATDELAKAM
0.221956	SIMDLENDKQQADEKIKKK
0.215944	GEVDRKLAEKDEEMEQIKRN
0.215371	AETLWGVQKELTL
0.212756	VTWYDNEFGYSNR
0.210672	MLELDGTENKSKFGAN
0.209331	SGDLEEELKNVTNN
0.208392	KNSYEESLDHL
0.208336	GEQIDNLQRVKQK
0.206164	LQSAETEKELANMK
0.206153	LNDQHLGKTK
0.202868	GTGEFSEPSVEIEPVEKL
0.201883	TADQIEDFKEAF
0.201552	NVLSGGTTMYPGIADRM
0.199498	VLGNSGLTDVIHM
0.199461	GVKGERGNSGPAGSAGSQ
0.196848	NSYEEALDHLETL
0.194672	ENDKQQADEKIKKKEFETT
0.191105	GTEFMQDPDVEHL
0.189142	LGQNPQNKEVAAI
0.188573	GARQQEGESRLN
0.188189	EAEASLEHEESKIL
0.18772	EYDEAGPSIVHR
0.186978	GQKDSYVGDEAQSKRG
0.183688	VEELKLFLQ
0.181834	ELLEGKVLPGVDAL
0.177117	ELKKEQDTSSHLERM
0.176656	LLQSAETEKELANMK
0.176592	GVNHEKYDNSLK
0.175952	NSYEEALDHL
0.175926	LSKVEDEQSLGAQL
0.174546	KDSYVGDEAQSKRG
0.174138	GADTSADLEETGRVL
0.173645	KNSYEEALDHL
0.173594	VGSQVSEANLR
0.171621	LVTWYDNEFGYSNR
0.170832	DTSADLEETGRVL
0.167041	TENGEFGRQL
0.166978	AEELKKEQDTSSHLERMKK
0.165729	SYVGDEAQSKRG
0.165635	IIDQNRDGIIS
0.162679	SGDLEEELKNVTN
0.158324	DTMDRIIQTM

0.155714	LAEKDEEMEQIKRN
0.155624	LRIDQSILTGE
0.155039	SESLKDAQEKL
0.154054	IAMDGTEGLVRGQ
0.148766	LQSAETEKELANM
0.146696	SKIEDEQSLGAQ
0.146162	VGMGQKDSYVGDEAQSKRG
0.145971	SKVEDEQSLGAQLQ
0.142977	WKQKYEEGQAELEGA
0.142884	ETLWGVQKELTL
0.1421	AKVIHDNYH
0.139815	EEAEGTLEHEEAKIL
0.136843	GVVGNAGEKGEQGPS
0.136801	GQKDSYVGDEAQSKR
0.135484	KVIHDNYH
0.132405	AEELKKEQDTSSHLERMKKN
0.131981	EVKKEVKEEEEVGDWRK
0.130324	GYTGATGATGLTGPAGPQ
0.130159	GVNHEKYDNSLKVVSN
0.129206	LENDKQQADEKIKKK
0.128406	NFSHGTHEYHAE
0.127608	LLEKEAADLITEK
0.127089	DLENDKQQADEKIKKK
0.126999	VDHVNKDIAAK
0.125857	VDRKLAEKDEEMEQIKRN
0.12519	LLSKVEDEQSLGAQ
0.123158	SKVEDEQSLGAQ
0.122966	AEWKQKYEEGQAELE
0.121666	VTGGKSNDDVSGPANQL
0.119758	PTETQSDARSF
0.117163	GVNHEKYDNSLKVVSNA
0.116583	TNEKLQQF
0.113098	TDAEQEQLIADHF
0.112937	LSKVEDEQSLGAQLQ
0.112822	EKDEEMEQIKRN
0.111447	KVEDEQSLGAQLQ
0.107434	EELEEAKKKLAQR
0.107353	EEMEQIKRN
0.104621	VVSSDFNGDTHSS
0.101896	GRTEHTPEGVKWVDTQ
0.101605	EEISERLEEAGGAT
0.101449	LIEDTQDWQPRTGTTQ
0.0990451	LSKVEDEQSLGAQ
0.0964986	GQKDSYVGDEAQSK
0.0945146	DISNADRLGSSEVDQVQ
0.0942321	IAEEADRKYEE
0.0937397	ITGESGAGKTVNTK
0.0932384	TLDDLQSEEDKVNTL
0.0923273	TLDDLQAEEDKVNTL
0.0860746	VGMGQKDSYVGDEAQSK
0.0858503	SDLERTEERAELSEGK
0.0847766	ELEEELKNVTNN
0.0839209	ISDLTEQIGETGKS
0.0833431	KLKGTEDELDKY
0.0823965	LQNEIENVKGQ
0.0793251	NDENVRQVNDISGQ
0.0744901	LEEELKNVTNN
0.0718552	EELEEAKKKLAQ
0.0715598	NADRLGSSEVDQVQ
0.0710519	ELKKEQDTSSHL
0.0704079	KRAIEEEVKAK

0.0702853	METGLTPDQVKKN
0.0688986	SELEEELKTVTN
0.0652111	DENVRQVNDISGQ
0.0651457	KTIDDLEDELYAQ
0.0646284	KTKNDENVRQVNDISGQ
0.0637462	LAKVEKEKHATEN
0.0625328	LIEEEKKEMKQE
0.0623686	ELEEELKTVTNN
0.0617897	ELEEELKNVTN
0.060841	EIQLSHSNRQAAEAQ
0.0600667	ENVRQVNDISGQ
0.0559723	TLENGNLVQKQ
0.0553383	KRQLEEEVKAK
0.0538266	TALEEAEGTLEHE
0.0528499	ELEEELKTVTN
0.0526873	TEQIGETGKSIHE
0.0524878	LEEELKTVTNN
0.0487858	LVEEELDRAQ
0.0467464	AYKRHSEEAEEAANQH
0.0393565	TKEKKALQEAHQQ
0.0344744	EKAKKTVETEKSEIQ
0.0310724	VEMEKKLEKGEAIDGMIPAQK
0.0287943	LKEVKKEVKEEE
